# The decrease in diurnal oxygen production in Elodea under the influence of high geomagnetic variability: the role of light, temperature and atmospheric pressure

**DOI:** 10.1007/s00484-023-02457-9

**Published:** 2023-03-27

**Authors:** Elizabeth Davies

**Affiliations:** Eastbourne, UK

**Keywords:** Geomagnetic disturbance, Photosynthesis, *Elodea*, Diurnal oxygen production, Zeitgeber, Metabolic depressant

## Abstract

Epidemiological studies have indicated adverse effects of geomagnetic disturbance on human health, including increased mortality. There is evidence from plant and animal studies that help to elucidate this interaction. This study tests the hypothesis that geomagnetic disturbance affects living systems, by modifying the metabolic process of photosynthesis, in the natural environment.

Continuous 24-h measurements of dissolved oxygen in flasks containing Holtfreiter’s solution and strands of healthy *Elodea* were recorded from May 1996, until September 1998, in an electromagnetically quiet, purpose built, garden shed environment, without mains electricity. Sensormeter recordings of oxygen, light, temperature and air pressure were uploaded weekly to a PC. The hourly total geomagnetic field measurements were obtained from the nearest observatory.

Significant decrease in oxygen (diurnal volume of oxygen divided by plant mass and diurnal light), (O/WL), was found on days of high geomagnetic field variability throughout 11 recorded months of the year 1997. This result was independent of temperature and atmospheric pressure. No significant decrease in O/WL during high geomagnetic variability was found for the 7 months recorded in 1996. The 1996 and 1997 data both showed a significant decrease in the diurnal time lag between peak light and peak oxygen for diurnal high geomagnetic variability compared with low geomagnetic variability. Cross correlation analysis for 1997 and 1998 data showed a decrease in positive correlation of oxygen with light in high geomagnetic variability, compared with low geomagnetic variability, and increased positive correlation with the geomagnetic field instead. These experiments support a hypothesis of high geomagnetic field variability as a weak zeitgeber, and a metabolic depressant for photosynthetic oxygen production in plants.

## Background

The geomagnetic field has a diurnal cycle, and the amplitude and rhythms of this cycle vary throughout the year, and over larger time scales, which include an 11-year cycle. It is a complex field, exhibiting a steady-state total field vector, with a gradient from 25,000 to 65,000 nanotesla across the globe, with micropulsations 1/10,000^th^ of the main field, which vary in frequency and amplitude. It has been suggested that the variability in the field correlates not only with movements in the Earth’s core and viscous mantle, with the seismic activity caused by stresses in rocks of the mantle and crust (Aubert et al. 2013; Straser 2012), and with water in its various states and gravimetric fluxes in and around the Earth (Barlow et al. 2013), but also with the ionospheric changes due to lightning and thunder storms, solar flares and cosmic rays (Gonzalez et al. 2011). Life on earth has evolved in phase with the dynamics of this geomagnetic field.

There has been growing evidence over the last seventy years for effects of the geomagnetic field (gmf) on animal and plant cell enzymatic activity (Krylov et al. 2019). This has involved such diverse systems as baroreceptor activation in human cardiac vascular system (Gmitrov 2007), gene expression in seed germination and development (Maffei 2014) and chemotaxis in slime moulds (Pazur et al. 2007). There have been many studies of adverse health effects in the human population, associated with geomagnetic storms (Zilli Vieira et al. 2019). There are many prevalent theories to explain the subtle and variable effects of geomagnetic field interaction with living cells (Pazur et al. 2007).

In plant cells, a complex ion transport system is involved in the photosynthetic pathway involving passage of electrons and protons across the thylakoid membranes. This is important in light dependent oxygen production, and can be modified by changes in gene expression (Agliassa et al. 2018). Proton precession changes in fluids are commonly used to monitor changes in the geomagnetic field (gmf) at the 0.1 nanotesla level. Proton precession magnetometers are used to record the diurnal gmf at observatories in the UK. It is considered possible that the geomagnetic field may affect plant oxygen production, by interaction with protons and proteins in the photosynthetic pathway. Laboratory experimentation has shown a simulated GMF influence on photo-morphogenic promoting gene expression in Arabidopsis seedlings (Agliassa et al. 2018). However, it is not known whether such GMF could influence measurable changes in oxygen production. The aim of this experiment is to monitor the influence of natural GMF on oxygen production by the freshwater pond weed *Elodea* sp., testing the hypothesis that changes in diurnal variability in the geomagnetic field affect diurnal oxygen production in plants. This may be the first study to test this hypothesis, using *Elodea*, a freshwater plant.

## Methods

The experiment was conducted, as planned, in an electromagnetically quiet environment, (no buildings within 50 m), in two dedicated wooden sheds with glass windows, and no mains electricity, at the bottom of a garden in a small village in S.E. England. Only battery operated equipment was used.

Fresh *Elodea* was obtained from a garden centre and grown in a tank outside the sheds, in the garden in a freshwater pond medium, called ‘Instant Pond’, based on Holtfreter’s solution, NaCl 0.059 M, KCl 0.00067 M, CaCl_2_ 0.00076 M, NaHCO_3_ 0.0024 M. A stalk with several strands of *Elodea* were placed in each flask containing solution. Although the strands were not uniform in size, dry weight measurement, after experimentation, allowed for specific oxygen calculation. The flasks were sealed by glass lids with holes to allow the electrodes to be fixed securely inside the flask for monitoring dissolved oxygen, temperature and light.

The datalogger, sensormeters and all electrodes were supplied by Philip Harris, a UK biological laboratory equipment company (philipharris.co.uk).

The oxygen sensormeter with its Clarke type Oxygen electrode was used for continuous monitoring of dissolved oxygen, with recordings at 23-min intervals, and a resolution of 0.5% full scale, and accuracy limited by state of electrode and daily calibration. The Clarke oxygen electrode has been used before, for monitoring oxygen in submerged plants (Sorrell and Dromgoole 1987; Sorrell and Armstrong 1994; Häder and Schäfer 1994). The data range was specified as most sensitive between 0 and 30%, with normal air oxygen content nominally 20%, a full-scale oxygen measurement of 100, on the sensormeter, the molar value of oxygen in air (which was equivalent to 4.4 mmol of dissolved oxygen in a litre flask).

A temperature probe connected to a temperature sensormeter was used in each flask. The oxygen data required adjustment for every degree of temperature change after calibration, ± 2.5%, according to manufacturer’s specification, subtracting for increase and adding for decrease. This was performed by using a macro to convert the uploaded data accordingly. The Temperature SensorMeter used a sensor range of − 20 to + 50 °C with a resolution of 0.5% full scale.

The light-level SensorMeter was used in a logarithmic range, covering the range of light levels from 1–100,000 lx with a resolution of ± 0.2% full scale, and spectral response of 400–1000 nm.

Atmospheric pressure was measured daily using the Air Pressure SensorMeter, with a range of measurement from 900 to 1100 millibars. The DLplus datalogger allowed simultaneous recording of 4 SensorMeters, with full battery operation and continuous recording for 7 days, at a rate of 2.6 samples hourly.

The recorded data was transferred, each week, to a PC and opened in an Excel spreadsheet. As this was an innovative and unfunded experiment, the equipment was chosen for its ease of use and affordability. The responsiveness and accuracy of the oxygen electrode was confirmed by using a known calibration curve of oxygen/glucose consumption by actively growing yeast.

Each night, the oxygen electrodes were checked and recalibrated in air over well-shaken water, according to the manufacturers’ instructions. The electrode membranes and solution were replaced if and when necessary, according to the manufacturers’ instructions. After 7 days, the *Elodea* was removed, blotted dry, and oven air-dried for 1 h, and weighed. The dry weight was recorded in grams. The relative volumes of oxygen and light were ascertained from graphical data plots, by cutting out the area under each parameter plot of light and oxygen, individually, and recording the weight of each in grams. The oxygen quantity thus obtained was divided by the light quantity and *Elodea* weight, giving a specific relative oxygen value O/WL, or relative oxygen volume per unit weight and light. This was an innovative method, designed for this research project, based on the principal of integration mathematics, or determination of the area under a curve.

Geomagnetism is monitored worldwide by a series of geomagnetic sensing stations. In the UK, there are three main stations, one at Eskdalemuir in Southern Scotland, one at Hartland Point in Devon, Southern England, and one in Lerwick, Northern Scotland. It was established, by use of a proton precession magnetometer (loaned by University of Brighton geology department), at the experimental location (latitude 50° 59′ N, longtitude 0° 8′ E) that the measurements locally followed the same diurnal variations as the nearest geomagnetic field observatory measurements. Therefore, diurnal hourly measurements of the geomagnetic field, total intensity (*F*) and the hourly mean standard deviations were obtained daily from the Hartland Point observatory, by courtesy of the British Geological Survey in Edinburgh.

The data was divided according to the variability in the geomagnetic field for each day, as determined by the diurnal mean standard deviation (sd) measured in nanotesla (nT). The data from 1996 was divided into two levels of gmf variability: gmf H (high gmf 7.5–22.72 nT) and gmf L (low gmf 1–6.7 nT). The data from 1997 was divided into 3 levels of gmf variability, gmf H (9nT-27nT), gmf L (1nT-9nT) and gmf M (2nT-26nT). The daily measurements of specific oxygen and total light were calculated, as described, and recorded alongside diurnal gmf sds, temperatures and atmospheric pressures, into an excel spread sheet (see supplementary data xls.1, also https://data.mendeley.com/datasets/669fyf6kxt/1).

Data from January during 1997, when the temperature was below zero, had to be rejected from the data sets, as table top paraffin heaters were placed under the flasks in the sheds to prevent the water freezing. This introduced unquantifiable, confounding variables, including extra CO_2_ in the immediate surroundings.

Two different electrode/*Elodea* set-ups were used in two simultaneous experiments each week. Both flasks were in the same position relative to window light, but as the sheds were set apart from each other, this meant a different light exposure profile for each set-up, and different electrode response for each. However, the specific relative oxygen parameter, O/WL, used in analysis, ensured parity between samples, with respect to any possible geomagnetic field effect. Therefore, in the analysis, the results from both electrode set-ups are pooled together. As two different electrodes (A and B) were used, an analysis was included to control for possible confounding effects of electrode bias (see dataset 6a in Results section).

Data analysis for 1996 and 1997 involved not only specific oxygen (O/WL), and log light (light), but also atmospheric pressure in millibars, (AP), temperature in °C (T), time gap between peak light and peak oxygen levels in hours (lag), (see supplementary data file, xlsx.1). The data analysed in 1998 used the raw data for oxygen (% oxygen), which was calibrated with the 100% reading as the normal volume in air, and the log light measurements (see supplementary data file, xlsx.2 and https://data.mendeley.com/datasets/669fyf6kxt/1). All geomagnetic field measurements (gmf) are mean diurnal standard deviation (sd) in nanotesla (nT).

For some specific analyses, the influence of environmental confounders was tested, by filtering and subdividing the datasets accordingly. These analyses are explained more fully in the results section.

Statistical analysis of the data was performed using XLstat software, and involved parametric and non-parametric Ttests, including effect sizes, and the alpha significance level was set at 0.05. Cross correlation was used for time series analyses. The analyses used are described in more detail in the results section.

## Results

1. The seasonal variation for high and low geomagnetic field variability and the corresponding range of values for specific oxygen

### Dataset 1

**Fig. 1:**
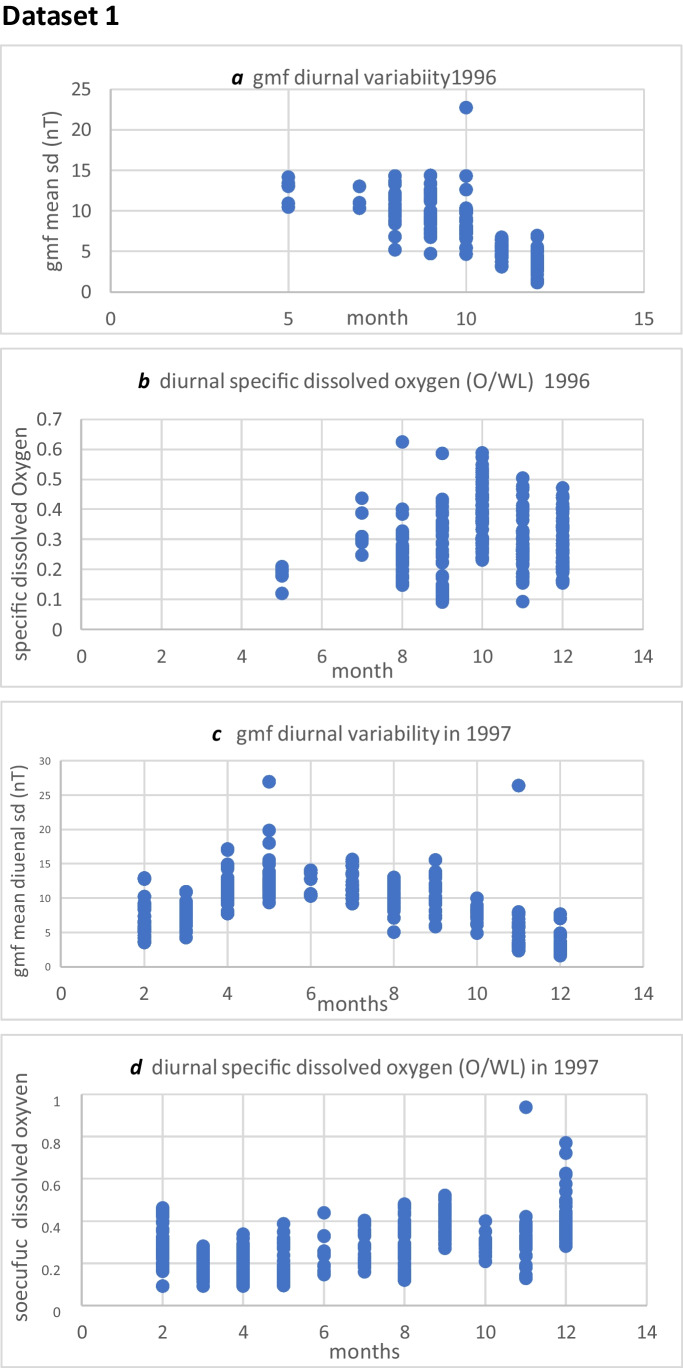
**a, b, c, d** showing the range of seasonal variation for gmf H and gmf L, and the corresponding range of values for O/WL, for each month in 1996 and 1997. 1996 O/WL( gmf H) 0.091–0.624; O/WL( gmf L) 0.094–0.588. 1997 O/WL (gmf H) 0.09–0.437; O/WL (gmf L) 0.11–0.939

The data from 1996 and 1997 showed a seasonal spread for gmf H and gmf L as shown in Fig. 1a,b,c and below*.* These figures also show the full range of specific oxygen values. These values are also shown in the raw data in the supplementary information files xls.1 and xls.2 https://data.mendeley.com/datasets/669fyf6kxt/1

2. High, medium and low geomagnetic field variability and the corresponding phase relationships of oxygen and light

### Dataset 2

**Fig. 2:**
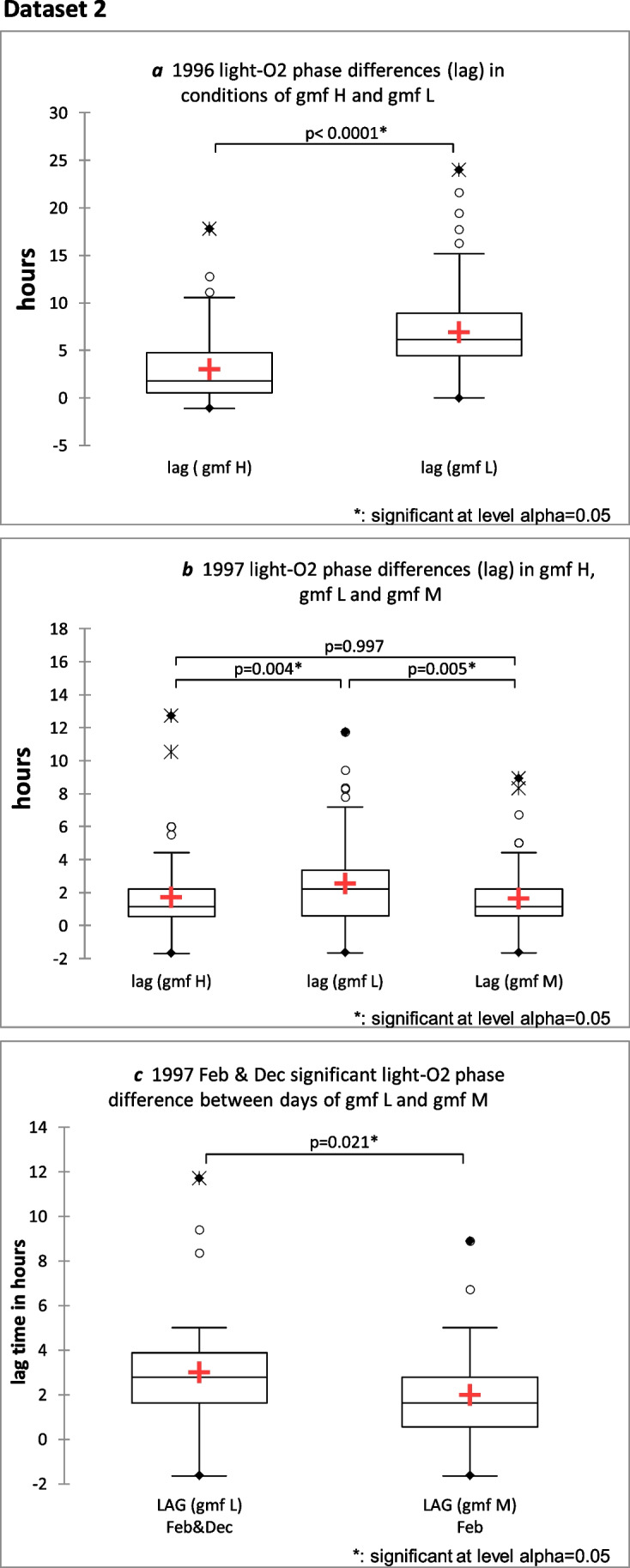
**a** Box Plot of Mann–Whitney two-tailed test, showing significant difference in LAG between light and O_2_ in conditions of gmf H and gmf L. (*P* < 0.0001, *n* = 155, effect size 1.1) in 1996. **b** Box plots of Kruskal–Wallis two-tailed test, showing significant difference in LAG between gmf H and gmf L (*P* = 0.004, *n* = 94, effect size 0.38); gmf L and gmf M (*P* = 0.005, *n* = 99, effect size 0.44) in 1997. **c** Box plot of Mann–Whitney 2-tailed test, showing significant decrease in LAG in gmf M during the cold winter months (*P* = 0.021, *n* = 40, effect size 0.45) Gmf L and gmf M, in 1997 cold winter months, showed a significant difference by Mann–Whitney two-tailed test (*P* = 0.0001, *n* = 40)

Statistical analysis of the data, in both 1996 and 1997, showed a significant difference in the phase relationship (LAG) of diurnal peak oxygen and light during the different conditions of gmf H and gmf L*. (P* < 0.0001, *n* = 155, effect size 1.1) and (*P* = 0.004, *n* = 94, effect size 0.38) respectively. Statistical analysis for 1997 also showed a significant difference in LAG for the different conditions of gmf L and gmf M (*P* = 0.005, *n* = 99, effect size 0.44). This shift is shown as a significant decrease in LAG during higher geomagnetic variability as shown in the Mann–Whitney two-tailed test (for two samples), for 1996 (Fig. 2a), and in the Kruksal-Wallis two-tailed test (for more than two samples), for 1997 (Fig. 2b). These comparable non-parametric tests were used, as some of the data did not show normal distribution. The significant difference in the GMF variabilities, gmf H and gmf L in 1996 data, by Mann–Whitney test is as follows: (*P* < 0.0001, *n* = 158, effect size 3). The significant difference for the GMF variabilities for gmf H, gmf L and gmf M in 1997 data is respectively*:* (*P* < 0.0001, *n* = 94, effect size 3.2; *P* < 0.0001, *n* = 94. Effect size 1.22; *P* < 0.0001, *n* = 101, effect size 1.66).

The data for 1996 comprised mainly the second half of the year (see Fig. 1a and b), gmf L (1–6.7nT), gmf H (7.5–22.7nT). The data for 1997 comprised almost the whole year (see Fig. 1c and d), gmf L (1–9nT), gmf M (2–26 nT), gmf H (9–27 nT).

For 1997, the majority of gmf L was in the colder winter months (88%) (see Figs. 1c and 1d and supplementary data file xls2 and https://data.mendeley.com/datasets/669fyf6kxt/1). The majority of gmf H was in the warmest months (100%); gmf M spanned both the colder and warmer months, with 40% in the colder months.

Therefore, to check for seasonal confounding influences on the significant LAG (phase shift) effect of gmf, a new analysis was made of data, using only winter samples for gmf L (December and February) and only the winter February samples for gmf M. This analysis showed a significant difference in LAG (phase shift) between the oxygen and light (*P* = 0.021, *n* = 39, effect size 0.45) at the 0.05 alpha level, during days of higher gmf variability (gmf M) and days of lower gmf variability (gmf L) (see Fig. 2c). For these winter months, the gmf M and gmf L difference is significant at the alpha level of 0.05 (*P* = 0.0001, *n* = 40, effect size 1.125).

This result suggests that gmf may have an effect on phase shift, independent of seasonality.

3. Correlation analysis of 1997/1998 time series data

### Dataset 3

In order to further understand the nature of the interactions between light and geomagnetic field and oxygen, cross correlation analysis was performed on the 1997/1998 data, using the hourly data for log light, % oxygen and gmf mean sd, from 21 days of gmf L and 21 days of gmf H. ***(see supplementary data file xls.1 and*** https://data.mendeley.com/datasets/669fyf6kxt/1***).***

**Fig. 3:**
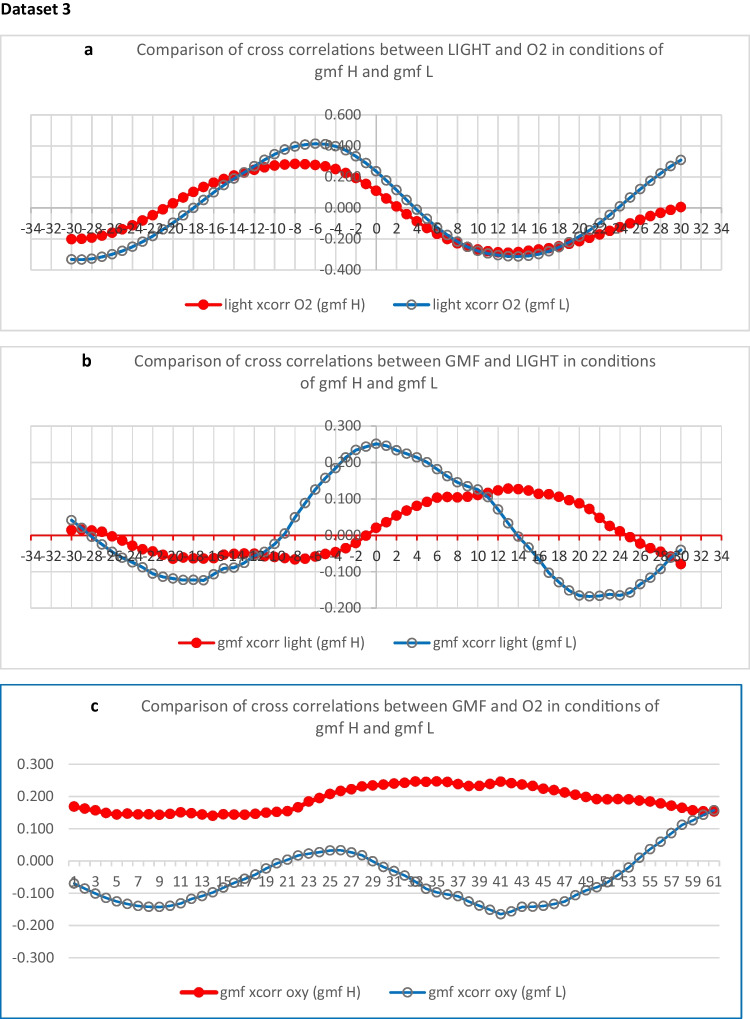
**a, b, and c** give graphical displays comparing correlation data for gmf H and gmf L conditions, during 21 days in 1997–1998. **a**, **b**, and **c** show, respectively, the differential effects of gmf variability on the diurnal time series interactions of O_2_ and light, (**a**) and gmf and light (**b**) and gmf and O_2_ (**c**)

It should be noted that in cross correlation analysis, lag at 0 suggests complete synchrony of two time series. Positive or negative lags from 1 onwards suggest that a level of synchrony is reached after the specified discrete phase time lapses, with one time series either ahead of or behind the other, according to the ± position on the horizontal axis. The series are either positively or negatively correlated, according to the value and plus or minus position on the vertical axis, where the degree of correlation is between 0 and ± 1 (0 being zero correlation and 1 being total correlation) (see following Figs. 3a,b and c).

Figure 3a suggests that oxygen and light are positively correlated at lag − 6, and that the level of correlation is diminished during gmf H, or high gmf variability. Figure 3b suggests that the positive correlation between gmf and light at lag 0, disappears during gmf H or high gmf variability. Figure 3c suggests that there is no correlation between gmf and oxygen during gmf L, but there is positive correlation at all lags during gmf H. The optimum light conditions for photosynthesis may be desynchronised from optimum oxygen production during gmf H, and gmf H may then become a weak zeitgeber for oxygen. The following analyses support this suggestion.

4. High, medium, and low geomagnetic variability and the corresponding effects on specific oxygen production in 1997 and 1996

### Dataset 4

The following analyses show the interaction of the variability in the gmf with the diurnal dissolved oxygen output from *Elodea*, using the specific oxygen (O/WL) parameter as an indicator of oxygen independent of quantities of light and plant material.

**Fig. 4:**
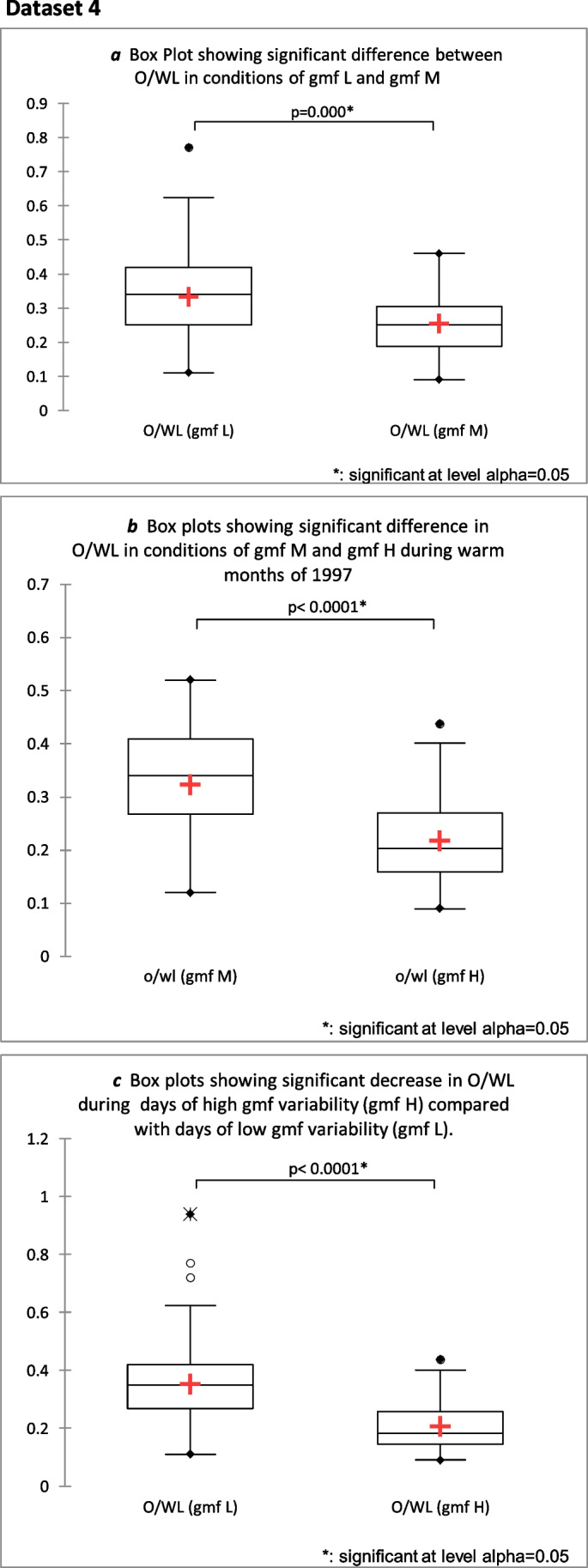
**a** Box plot of Mann–Whitney 2-tailed test showing significant difference (*P* < 0.0001, *n* = 71, effect size 0.55) between O/WL in conditions of gmf M and gmf L during the colder months of 1997. Significant difference between gmf M and gmf L in 1997 during colder months of 1997, was shown by Mann–Whitney 2-tailed test (*p* < 0.0001, *n* = 72)**. b** Box plot of Mann–Whitney two-tailed test: showing significant difference in O/WL during gmf M and gmf H (*P* < 0.0001, *n* = 72, at alpha significance level 0.05, effect size 1.1) during warm months of 1997. Significant difference between gmf M and gmf H, during the warmer months of 1997, was shown by Mann–Whitney 2-tailed test (*P* < 0.0001, n = 74). **c** Box plot of Mann–Whitney test: showing significant difference in O/WL in conditions of gmf H and gmf L in 1997 (*P* < 0.0001, *n* = 94, effect size 1.3) for data spanning all seasons. The difference between gmf L and gmf H was significant by Mann–Whitney 2-tailed test (*P* < 0.0001, *n* = 92, effect size 3.1)

The data for 1997 was divided into gmf L, gmf M and gmf H. The analyses show***:*** (Fig. 4a) significant decrease in O/WL during gmf M compared with gmf L (*P* < 0.00014, *n* = 71, effect size 0.55) (Fig. 4b) significant decrease in O/WL during gmf H compared with gmf M (*P* < 0.0001, *n* = 74, effect size 1.1) (Fig. 4c) significant decrease in O/WL during gmf H compared with gmf L (*p* < 0.0001, *n* = 100, effect size 1.3), for data spanning all seasons. Significance level was set at 0.05 for all Mann–Whitney tests (see following Figs. 4a,b, and c).

### Dataset 4a

In this 1997 dataset, samples were taken only from the colder months, showing non-significant temperature difference between the samples in gmf L and gmf M, as shown by Mann–Whitney two-tailed test (*P* = 0.07, effect size 0.15, at alpha significance level of 0.05). However, there is significant decrease in O/WL (*P* = 0.00014, *n* = 74, effect size 1.37) (see Fig. 4a).

### Dataset 4b

In the following 1997 dataset, O/WL samples were taken from the warmer months, exclusively, with no significant difference in temperature (*P* = 0.835, at alpha significance level 0.05, effect size 0.06) between samples in conditions of gmf M and gmf H. Mann–Whitney two-tailed analysis shows a significant decrease in O/WL during gmf H compared with O/WL during gmf M (*P* < 0.0001, *n* = 72, at alpha significance level 0.05, effect size 1.1) (see Fig. 4b).

### Dataset 4c

In the following dataset, O/WL samples were taken from the whole seasonal span, and showed a significant difference in O/WL (*p* < 0.0001, *n* = 100, effect size 1.3) during the two conditions of gmf L and gmf H. The difference between gmf L and gmf H was significant by Mann–Whitney 2-tailed test (*P* < 0.0001, *n* = 92, effect size 3.1) (see Fig. 4c).

### Dataset 4d

The data for 1996 includes 7 months (as shown in Figs. 1a and 1b), and involves only 161 days, and 280 complete experimental data series. This data does not show a significant decrease in O/WL during gmf H (*P* = 0.337, *n* = 140, effect size 0.07) despite a significant difference between gmf H and gmf L (*P* < 0.0001, *n* = 140). There were significant differences in both temperature (*P* < 0.0001) and atmospheric pressure (*P* = 0.001) in the conditions of gmf H and gmf L.

Two of the previous dataset results (datasets 4a and 4b) suggest that temperature is not a determinant for the significant decrease in O/WL, shown in the results of dataset 4c, during conditions of gmf H. However, atmospheric pressure (AP) had been found to be significantly different in dataset 4a (*P* = 0.004, effect size 0.67), dataset 4b (*P* = 0.061, *n* = 74, effect size 0.34) and dataset 4c (*P* = 0.001, effect size 0.4). Therefore, it was necessary to check whether AP rather than gmf was a determinant in the significant decrease observed in O/WL in datasets 4a,4b and 4c.

5. High and low atmospheric pressure and effect on specific oxygen production

### Dataset 5a

**Fig. 5:**
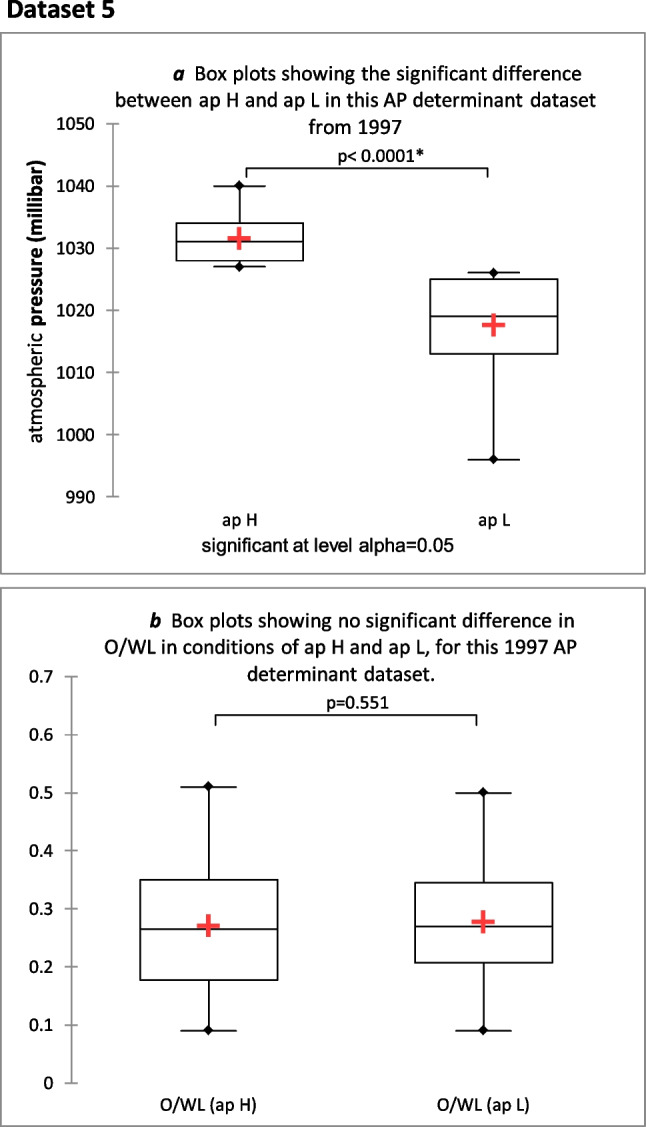
**a** Box plot of Mann–Whitney 2-tailed test: showing significant difference between ap H and ap L (*P* = 0.0001, *n* = 101, effect size 2.5) in this 1997 AP determinant dataset. **b** Box plot of Mann–Whitney test: box plot showing no significant difference between O/WL in conditions of ap H and ap L (*P* = 0.551, *n* = 101, effect size 0.06)

For the following analysis, the 1997 dataset was divided into two parts according to atmospheric pressure levels, higher (above 1027 mbar) and lower (below 1027 mbar) (ap H and ap L respectively) using AP as the determinan. (see Fig. 5a). No significant difference was seen in O/WL in conditions of ap H and ap L (*P* = 0.551, *n* = 101, effect size 0.06) (see Fig. 5b). No significant difference was seen in the gmf between conditions of ap H and ap L (*P* = 0.06, *n* = 101, effect size 0.22).

6. Electrode specific effect and corresponding changes in O/WL in conditions of gmf H and gmf L

### Dataset 6a

**Fig. 6:**
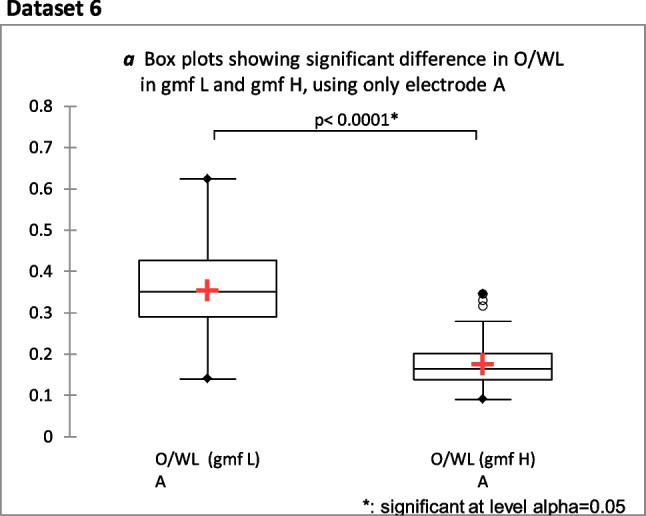
Box plot of Mann–Whitney 2-tailed test: showing significant decrease In O/WL in gmf H compared with O/WL in gmf L (*P* < 0.0001, *n* = 63, effect size 2.3) for all seasons in 1997 using only electrode A

As two different electrodes were used in experiments, an analysis was included to control for possible confounding effects of electrode bias. The following results were obtained, using a dataset with samples exclusively from experiments performed with electrode A, to control for any possible confounding effects of data skewing, caused by differential electrode characteristics. The significant decrease in O/WL during gmf H conditions is further supported in this analysis (*P* = 0.0001, *n* = 62, effect size 2.3) (see Fig. 6a). There was significant difference between gmf H and gmf L in this analysis (*P* < 0.0001, *n* = 63, effect size 2.9).

## Discussion

The 1997 and 1996 data both support the hypothesis of gmf H as a weak zeitgeber, by showing a significant difference in time lag between O/WL and light during photosynthesis. The 1997 data shows a significant decrease in O/WL, during diurnal gmf H, independent of temperature and atmospheric pressure, supporting the hypothesis of gmf H as a damping factor in oxygen production in *Elodea*. However, the smaller dataset from 1996 does not fully support the latter hypothesis, as no significant decrease in O/WL was shown.

There are some possible explanations for the difference in these results between 1996 and 1997. The actual species of the genus *Elodea* was not specified in these experiments. The *Elodea* in 1996 was from a different garden centre than the *Elodea* in 1997.

Jones et al (2000) found no significant difference between two species of *Elodea, E. nuttella* and *E. canadensis*, except at low pH(< pH6), with regard to oxygen production under specified environmental conditions*. E. nuttella and E. canadensis* and *E. densa* are the three most common species in the UK, originating from the Americas. Rakosy-Tican et al. (2005) showed, experimentally, that magnetic field exposure effects on plant growth and development was either stimulating or inhibiting, dependent on species, genotype, treatment duration and culture medium.

The lack of plant specification in these experiments may mean that the metabolic status and health of the *Elodea* used in 1996 was different from that used in 1997, and could have been responsible for a differential response to gmf H.

Shine et al. (2012) showed that plants can perceive and respond quickly to varying magnetic field, by altering their gene expression and phenotype. Therefore, it seems possible that specific features of the geomagnetic field in 1997 might have triggered phenotypic changes, especially with samples growing during the peak growth periods of early spring, which is when the *Elodea* source changed. Peak growth months were only monitored in 1997.

Another possible difference between 1996 sampling and 1997 was the environmental factor of peak light values, and spectral differences in the light due to seasonal, solar and ionospheric influences. With regard to peak light, the light levels experienced by *Elodea* in the flasks were below the light levels considered sufficient to trigger the oxygen saturation negative feedback response. Jones et al. (2000) showed that light over 290 umols/m2/s (over 4.079 log lux) were needed to cause photorespiration to overtake photosynthesis (Jones et al. 2000), as oxygen surfeit has been shown to cause preferential competitive binding of oxygen, rather than carbon dioxide, to a key enzyme in the photosynthetic/carbon fixation pathway, ribulose diphosphate *carboxylase*, known as Rubisco (Lorimer 1981). Peak light level in the flasks for both years surpassed this level for 2.9% of samples in 1996, and 1.9% of samples in 1997. In 1996, 7 months of the year were sampled, and in 1997, 11 months of the year. In 1996, out of 273 samples, 20.6% had peak oxygen values over 100%. In 1997, out of 418 samples, 24% had peak oxygen values over 100%. It is unknown, but unlikely, whether oxygen production reached a steady state of quenching, as the oxygen electrode was only calibrated up to 100% (4.4 millimols in a litre flask) and only allowed for an overshoot up to 120%. (On a hot day, the level would be 0.25 millimols in a litre flask).

Considering these facts, it does not seem likely that light levels would have been responsible for the apparent different O/WL response to geomagnetic variability between 1996 and 1997. However, the different spectral properties of the light between the sampling groups for 1996 and 1997 may have contributed to this effect. There were more samples in the warmer months in 1997 than in 1996 (see Fig. 1a–d). In these earlier spring and summer seasons, the plants are growing and their chloroplast expression and light-harvesting properties may be very different from those in the later months of the year. In 1996, the majority of samples were in the autumn and winter months. The 1997 data shows that a greater level of significant decrease is found in the warmer spring and summer months of the year, than in the colder autumn and winter months, (effect sizes, respectively: 1.1 and 0.55) (see Figs. 4a and 4b). A confounding environmental factor which might have accounted for a difference between O/WL response to gmf variability between 1996 and 1997, and was spectral properties in the light. Unfortunately, this factor was not monitored.pH in the surrounding medium was not monitored continuously in these experiments. It was seen to vary from 7 in the outside tanks, to 8 in the monitoring flasks. But it was considered unlikely that this was a significant factor, as *Elodea* has been shown to have a wide pH tolerance above pH 5 (Jones et al 2000).

Peak light values during 1997 correlated with a significant decrease in O/WL mean values at peak light > 3.34 loglux compared with O/WL mean values at peak light < 3.34, during conditions of gmf H and gmf M (*P* = 0.016 and *P* = 0.0001, respectively, by Mann–Whitney two-tailed test). There was no significant difference in O/WL mean values between 1996 and 1997 (*P* = 0.176)*.* This, along with the significant phase shift between light and oxygen in the two conditions of gmf H and gmf L (see Figs. 2a and 2b) suggest that the interaction of light with oxygen production is the sensitive parameter in the damping effect of gmf on O/WL.

The key to this may lie in photosynthetic activities in the thylakoids, and the complex synchronisations of the time constants in nonlinear activations of enzyme protein binding associations and dissociations. These are parameters that have been shown to be sensitive to electromagnetic disturbance (Eichwald and Wallaczek 1996; Gerardi et al 2008; Bareus Koch et al 2003)*.* The particular parameters of the electromagnetic signals involved in such disturbance may be relevant to the interaction of the gmf with O/WL.

The different response of *Elodea* between 1996 and 1997 may lie in differential characteristics of the gmf. There is evidence in the frequency analysis of geomagnetic diurnal time series, to show that every day has different harmonic characteristics, in amplitudes and pattern (Werneck de Carvalho et al 2015). Harmonic features in the gmf may resonate with specific electrochemical signalling in the thylakoid membranes, creating disturbances or alterations in time constants of enzymatic activity (Davies et al 1999). Examination of the data from the British Geological Survey’s 1996 and 1997 yearbook (British Geological Survey) show that there were 17 SSCs (geomagnetic storm sudden commencements) and SI (sudden impulses) during 1996, compared with 38 in 1997. A 10.9% of the sampling days in 1997 were exposed to these special gmf parameters, compared with only 3.9% of sampling days in 1996.

The signal specific details of the SSC and SI is interesting biologically. There is a sudden steep rise and reversal of polarity in the signal. The pulse period is 0.15–5 s, or frequency 0.2–6.5 Hz. This signal is characterised as the PC1 type pulse, which is usually a very low amplitude, continuous component of the gmf, in the picotesla range. However, a sudden increase in amplitude and reversal, even at this level, can be very meaningful in biological systems. The cardiac cycle, seen in the ECG, shows signalling with time constants in a similar range of 3.6 s (high-frequency component, 0.15–0.4 Hz). The biphasic signal used in defibrillation to restart a malfunctioning heart is a sharp electromagnetic pulse of similar characteristics to the SSC and SI seen in geomagnetic storms, but also in normal diurnal PC1 micropulsations at lower amplitudes (Natale et al 1995).

Magnetic fields can penetrate biological tissues, with no distortion and boundary effects. Cell membranes can act differentially, like tuned circuits in electronics, with resistive and capacitative effects, mediated by enzymes and ions, such as ionised forms of calcium, oxygen and carbon dioxide (Charman 2002)*.* In such circuits, the shape of the incoming signal, composed of harmonics with different frequencies and amplitudes, can create resonance and amplification of the natural and optimum performance or time constant of the circuit, or damp the peak activation and response. A very sharp rise time, and slow decay and reversal, has been suggested to be the effective agent in the use of pulsed magnetic fields to stimulate bone repair and wound healing (Charman 2002; Pilla 2020).

It seems that the important evolution of oxygen in photosynthesis is characterised by chains of molecular reactions from the femtosecond to the millisecond range, and even the subminutan range of seconds (Fleming and van Grondelle 1997; Haberkorn and Michel-Beyerle 1979; Ritz et al 2000; Kaila 2021). Protein conformational changes, which are an essential feature of the photosynthetic pathway, are shown to be in the millisecond range. Such changes are part of the bi-functional enzyme, Rubisco (Lorimer 1981), which can flip from photosynthetic pathway under the activation of carbon dioxide, to photorespiration pathway, under the negative feedback activation of oxygen, like a thermostat. The timing of all the binding and unbinding, association and dissociation constants involved are cooperatively interlinked, electromagnetically, by ionic charge transfer, and any shift in the timing, due to magnetic signal interference, can affect the longer term reaction rate of the whole process (Binhi and Prato 2018*).*

It has been shown how a sharp pulse of magnetic energy can help to reboot the neurological activity of the brain (Ye and Kaszuba 2019) in epilepsy, and reboot the growth of bone cells in osteoporosis (Shupak et al 2003). Admittedly, these are high energetic pulses that are used, much stronger than the geomagnetic field pulsations. But specificity of a resonant signal parameter, even in a white noise environment, can trigger amplification in a tuned circuit, especially in very nonlinear systems, where an initially small disturbance can have large effects further down the time line, as the metabolic process continues. Gene expression of proteins in the photosynthetic pathway has been shown to be affected by geomagnetic level fields in Arabdopsis (Agliassa et al 2018).

The light-triggered electrical events in the thylakoid membrane of plant chloroplasts has been characterised in its membrane voltage potential changes, using microelectrode patch clamping techniques. Bulychev and Vredenberg (1999) have shown how cytochrome b6f complex, which is the redox link between the two photosystems has electrogenic potential which directly affects changes in the photosynthetic membranes. The signal voltage change across the thylakoid membrane, when triggered by a pulse of light was measured as 70 mV biphasic pulse, with a sharp rise time of 20–50 ms, and slower decay of a second in the first phase, with a sharp negative drop on switching off the light, and slower return to base line, over a period of less than a second. The induced current ranged from − 0.4 to − 0.1 nanoAmps, and the membrane capacitance, was 1uF/cm^2^. The rise time of electro-stimulated fluorescence change in the photosystem cytochrome complexes of the chloroplast is shown to be similar to the time constant of the thylakoid membrane potential changes. These membrane voltage changes are always changing, with the light-level changes, and exert a control on the activity of important membrane enzymes, such as H + ATPase (Blackman et al 1994).

Paramagnetic oxygen and diamagnetic carbon dioxide competitively bind to Rubisco, in the first steps of carbon fixation in photosynthetic activity. They are key components of the negative feedback loop that controls photosynthesis and photorespiration. It has been shown that the geomagnetic field can impact on photoreception in Arabidopsis, and does not depend on light for this (Agliassa et al 2018).

Ion resonance theories have been proposed to explain the interaction of magnetic fields with living systems (Blackman et al 1994; Pazur 2004). These theories suggest a cyclotron resonant frequency for calcium ion of 31.6 Hz (Fitzsimmons et al1994; Nedukha et al 2007). The ion parametric resonance theory suggests a resonant frequency for the hydrogen ion at 45 Hz, with a bandwidth of from 40 to 50 Hz (Blackman et al 1999). Both these theories have been tested, and there is support for both hypotheses. Both the hydrogen ion and the calcium ion are very important in mediating protein folding and membrane enzyme transport. The time constants of these reactions are from microsecond to over 1 s, timescales, i.e. from megahertz to milliherz frequency. The SSC and SI characteristics of the diurnal geomagnetic field are sudden biphasic pulsations in the nanotesla amplitude region, ranging in amplitude from ± 3–46 nT in the horizontal (H) and ± 1–9 nt in the vertical (*Z*) vector of the GMF, considered to be connected with the solar wind. These signals have been shown by Clilverd et al. (2020) to produce harmonic currents in the power lines, distorting the 50 Hz fundamental (Clilverd et al 2020). The highest induced current frequency observed was 125 mHz and the lowest was 0.2 mHz.

The SSC and SI occurrence shows a difference between 1996 and 1997. Whether this, in itself, is a sufficient influencing factor in the differential response to GMF with respect to significant damping of O/WL, is highly speculative. However, the sudden sharp reversals in micropulsation are observed locally, using a Bartington 3-axial Fluxgate Magnetometer, and have been identified in the pc3 and pc4 region, at 10–20-s intervals (100–50 mHz), which is near the resolution limit of this particular local monitoring. These sharp reversals are quite a frequent component of the local GMF. Every diurnal GMF has been shown to have a different harmonic content. It was shown by Clilverd et al. (2020) that different harmonics at different amplitudes were noted at the same time, in different locations, and led to a different geomagnetic induced current (GIC) harmonic distortion profiles in different powerlines. Presumably for the same powerline, the distortion will be different on different days and throughout different years.

The pathways through which the geomagnetic field interacts with biological systems is an issue for ongoing research. The fact that it does, indeed, interacts with biological systems has been supported in recent years by research from many sources. There is increasing awareness of the dynamic interconnectivity of cosmic, terrestrial and biological fields: ionic, barometric, gravimetric, electromagnetic and associated rhythmic phenomena (Hunting et al 2021). Modern technology has introduced an electromagnetic environment that is complex and different from the natural electromagnetic environment in which organic life developed, and in which it has been sustained. Just as it seems that oxygen production in *Elodea* is depressed during naturally occurring minute changes in the geomagnetic field amplitude, so it seems there is depression of barometric cell responses in the heart system (Gmitrov 2005). If such small changes in a naturally occurring magnetic field, induce such important biological changes, how may the stronger fields that continuously surround our living environment for the last 100 years, be interacting with homeostasis?

## Conclusion

The data from these experiments support the hypothesis that increased geomagnetic variability affects metabolic process. Photosynthetic activity was shown to decrease during increased variability in the geomagnetic field. The data showed significant difference in the phase relationship of peak light and peak oxygen on days of higher compared with lower geomagnetic field variability independent of temperature, season and atmospheric pressure. This supports the hypothesis of high geomagnetic field variability, as a damping factor in oxygen production in *Elodea*. However, the smaller dataset from 1996 does not fully support the latter hypothesis, as a non-significant decrease in O/WL was shown. Some possible explanations for this difference have been suggested, involving specific features of the geomagnetic frequency spectrum and resonance with enzyme time constants in photosynthetic pathways. This could be confirmed by further experimentation.

These findings support an already established body of evidence, in many fields of research, including public health, suggesting an interaction of geomagnetic field with metabolic process.

## Data Availability

Data available https://data.mendeley.com/datasets/669fyf6kxt/1*).*

## Abbreviations

gmf: Geomagnetic field
sd: Standard deviation
gmf H: High geomagnetic field variability
gmf L: Low geomagnetic field variability
O/WL: Specific oxygen (oxygen diurnal volume divided by plant mass and light diurnal volume)
ap: Atmospheric pressure
ap H: High atmospheric pressure
ap L: Low atmospheric pressure
T^0^C: Temperature in degrees centigrade
SSC: Sudden storm commencement
SI: Sudden impulse
GIC: Geomagnetic induced current
LAG: Phase shift
